# Evolution of cardiorespiratory interactions with age

**DOI:** 10.1098/rsta.2011.0622

**Published:** 2013-08-28

**Authors:** D. Iatsenko, A. Bernjak, T. Stankovski, Y. Shiogai, P. J. Owen-Lynch, P. B. M. Clarkson, P. V. E. McClintock, A. Stefanovska

**Affiliations:** 1 Department of Physics, Lancaster University, Lancaster LA1 4YB, UK; 2 Faculty of Electrical Engineering, University of Ljubljana, Ljubljana, Slovenia; 3 Division of Biomedical and Life Sciences, Faculty of Health and Medicine, Lancaster University, Lancaster LA1 4YQ, UK; 4 Cardiology Department, Raigmore Hospital, Old Perth Road, Inverness IV2 3UJ, UK

**Keywords:** coupled oscillators, coupling function, ageing, cardiorespiratory interactions, phase coherence, synchronization

## Abstract

We describe an analysis of cardiac and respiratory time series recorded from 189 subjects of both genders aged 16–90. By application of the synchrosqueezed wavelet transform, we extract the respiratory and cardiac frequencies and phases with better time resolution than is possible with the marked events procedure. By treating the heart and respiration as coupled oscillators, we then apply a method based on Bayesian inference to find the underlying coupling parameters and their time dependence, deriving from them measures such as synchronization, coupling directionality and the relative contributions of different mechanisms. We report a detailed analysis of the reconstructed cardiorespiratory coupling function, its time evolution and age dependence. We show that the direct and indirect respiratory modulations of the heart rate both decrease with age, and that the cardiorespiratory coupling becomes less stable and more time-variable.

## 1. Introduction

The cardiorespiratory interaction was first observed by Hales [1]. Since then, the associated physiology and physics have been under continuing investigation. The mechanisms are still subject to debate [2], with the two prevailing approaches focused mainly on the heart rate variability (HRV) that occurs as a consequence of the interaction. One approach supposes that baroreflex plays a key role [3,4], whereas the other is based on the hypothesis of sympathetic/parasympathetic balance [5].

In this work, we adopt a different perspective. We treat the cardiac and respiratory functions as being oscillatory and interacting [6], and we consider the coupling to be enabled by two distinct mechanisms. The first is via the vasculature and the flow of blood through the system of closed tubes that connects the heart and the lungs. The second coupling occurs via the neuronal control that provides information transmission between all of the systems involved, i.e. the heart, lungs and vasculature. The oscillatory processes associated with the coupling mechanisms have been identified in earlier studies, and are related to endothelial [7–10], neurogenic [9,11,12] and myogenic [13,14] activity. We measure the oscillatory functions of heart and respiration, and then apply methods drawn from nonlinear science to detect their instantaneous phases and frequencies. Once these have been established, we study the interaction between heart and respiration, treating them as self-sustained nonlinear oscillators and investigating their mutual coordination [15–17] or synchronization [18–26], coherence [27–32] and direction of coupling [33–36], allowing for the fact that the characteristic frequencies, couplings and other interacting characteristics vary in time [37,38]. We report below an analysis of the results obtained from a study of 189 (99 male, 90 female) healthy subjects aged 16–90, enabling us to characterize the changes with age that occur in the cardiorespiratory interaction.

In §2, we present the methods of phase and frequency extraction used in the study, and we show that the synchrosqueezed wavelet transform (SWT) [39] offers significant advantages. In §3, we apply wavelet phase coherence (WPC) to investigate the components modulating the heart and respiratory rates. We formulate a model to describe cardiorespiratory interactions in §4. In §5, we extract the time-varying parameters of the model using recently developed methods based on Bayesian inference, and we investigate the age dependence of the derived measures: the directionality of coupling, synchronization and the overall coupling function. Finally, in §6, we summarize and draw conclusions. Details of subjects, measurements, signal pre-processing and statistical analyses are given in the appendices.

## 2. Phase and frequency extraction

To obtain the phase and frequency [40] of the respiratory and cardiac signals, we used the recently developed SWT [39]. The SWT *T*_*f*_(*ω*,*t*) is constructed from the wavelet transform (WT) *W*_*f*_(*a*,*t*) of the signal as
2.1
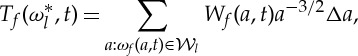
where 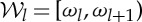 specifies a frequency bin of central frequency 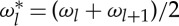 and width *Δω*_*l*_=*ω*_*l*+1_−*ω*_*l*_. For each wavelet scale *a* and time *t*_*i*_, we calculate the corresponding frequency *ω*_*f*_ as *ω*_*f*_(*a*,*t*_*i*_)=[arg(*W*_*f*_(*a*,*t*_*i*+1_))−arg(*W*_*f*_(*a*,*t*_*i*−1_))]/(2*π*(*t*_*i*+1_−*t*_*i*−1_)), where arg denotes the phase of the complex coefficient, and division by 2*π* is needed to go from circular frequency to the natural frequency in hertz. The sum in (2.1) is over scales *a* such that the calculated *ω*_*f*_(*a*,*t*) lies in the frequency bin 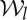 at time *t*.

The WT *W*_*f*_(*a*,*t*) is calculated using the Morlet mother wavelet [41, p. 70], and we use scales *a*_*j*_=2^*j*/*n*_*v*_^(2/*f*_s_), such that each dyadic interval is divided into *n*_*v*_ equi-log-spaced bins (*n*_*v*_ is chosen such as to provide appropriate numerical resolution; we use *n*_*v*_=64). In the latter expression, *f*_s_ denotes the sampling frequency and *j* is such that *a*_*j*_ ranges from 2/*f*_s_ to *T*/8.6 (so there should be at least eight oscillations for us to regard the apparent wavelet component as being physically meaningful), with *T* being the overall time of the signal. For the SWT (2.1), we use similar equi-log-spaced bin edges *ω*_*l*_ whose spacings are determined by the same *n*_*v*_=64, although we now investigate only the frequency region within which the corresponding first harmonics reside: [0.4,2.1] and [0.05,0.7] Hz for the electrocardiogram (ECG) and respiratory signals, respectively.

The advantage of the SWT over the WT is that it is far more compressed than the WT, which enables one to distinguish more accurately between different components in the signal. It becomes possible to select only one component (e.g. the main oscillation) and to filter out the others (e.g. higher harmonics). The signal *s*(*t*) can then be reconstructed from its SWT as 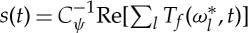, where *C*_*ψ*_ is a normalization constant [39].

We follow the support of the main oscillation (e.g. the first harmonic of the ECG), selecting a region of width *n*_*v*_/2=32 in frequency bins around it, adjusted so as to contain only the selected curve and no others. This enables us to extract the phase *ϕ*(*t*) and frequency *f*(*t*) as
2.2
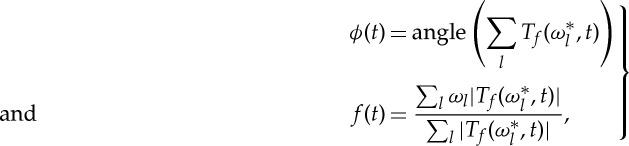
where the sums in (2.2) correspond only to the selected region. Extracting the frequency from the whole region proved to be more accurate than just taking it to be the centre of the main curve's support, because the SWT curves are often non-uniformly broadened.

**Figure 1.**
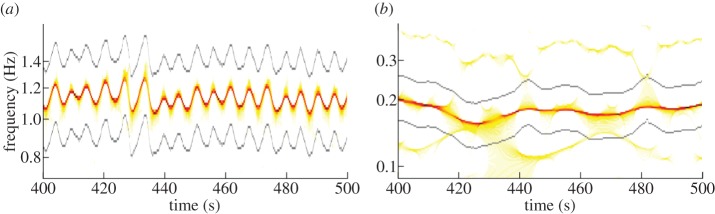
Synchrosqueezed wavelet transforms of (*a*) the ECG and (*b*) respiration. The support of the main curve, i.e. the selected region, from which phase and frequency were extracted, is shown by the grey lines. The oscillations in the ECG curve are at the respiration frequency and correspond to respiratory sinus arrhythmia. (Online version in colour.)

The main difficulty lies in automatically tracking the support of the main curve. At time *t*, we define it to be the region of width *n*_*v*_/2 having the maximum functional 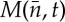, with 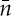 denoting its central bin:
2.3
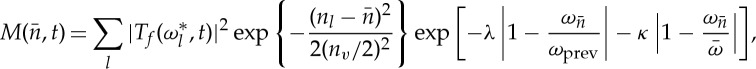
where 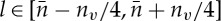. Here, 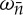 is the central frequency of the current window, *ω*_prev_ is the frequency (as in (2.2)) of the previous window (the corresponding term is used to suppress jumps) and 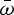 is the mean frequency, extracted as in (2.2), but over the whole range of *ω* (the corresponding term is used to give more stability to the main curve). The Gaussian term is needed to give more weight to the central part of the window, i.e. if there are two or more peaks in one window, to select the support of one of them. Despite its complexity, the method provides for good flexibility and the possibility of accurate tuning. Based on the characteristic variations of the fundamental frequencies, the optimal parameters were determined to be *λ*=5,*κ*=0 for ECG and *λ*=10,*κ*=10 for respiration. Figure 1 shows examples of the extracted supports of the SWT curves corresponding to the ECG and respiration first harmonics.

The advantage of using SWT for phase and frequency extraction over the more commonly used marked events (MEs) method is that one obtains phase and frequency values at the same sampling frequency as the signal, whereas the ME method gives values only at particular points (e.g. peaks). Thus, the effective sampling frequency of the characteristics extracted by MEs is, in fact, the (time-varying) frequency at which the MEs occur, i.e. the reciprocal of the current interpeak interval (usually around 1 Hz for ECG and 0.25 Hz for respiration); all other values, obtained by interpolation to match the original sampling frequency, are unphysical.

Note that heart activity is sometimes modelled through use of the integral pulse frequency modulation (IPFM) model [42] which, in effect, assumes that only the R-peak positions in the ECG are informative. Within this framework, much effort has been directed towards the reconstruction of instantaneous heart frequency (IHF), treated as the input signal. These methods involve the analysis of an RR interval series or a pulse-train equivalent of the ECG [43], from which the continuous IHF is obtained by low-pass filtering, spline interpolation or other techniques [42,44]. With such an approach, the interbeat information is necessarily neglected, so that the IHF cannot be obtained with a physical sampling frequency higher than that at which the R-peaks occur. In this sense, such methods can be regarded as variations on the ME method, but with different implicit or explicit interpolation schemes. They provide a very good approximation within the assumption that IHF modulation can be represented by a few sinusoids of constant amplitude and frequencies no higher than half of the mean heart frequency [44–46]. However, there exist many input signals that can give the same peak positions, so one cannot say anything for sure about the IHF between peaks without also taking account of interbeat behaviour. By contrast, the SWT uses all of the information, in effect yielding the IHF with a high effective sampling frequency. For example, in the case when all RR-intervals are the same, but the T-wave occurs at different times after the R-peak, methods based on the IPFM model assumption will yield a constant heart frequency, whereas the IHF extracted from the SWT will be slightly modulated, thus providing an indication of these small changes. Additionally, SWT frequency extraction does not require a high ECG sampling frequency, in contrast to the above-mentioned methods [47]. It is also more universal in that it is applicable to almost any oscillatory signal and not only to series of point events.

The advantage of the phase extracted from SWT over the commonly used Hilbert phase is that the former can be already regarded as a true phase, whereas the latter is a protophase (see [40] for definitions and a discussion of the differences). This is because the SWT phase is extracted from the fundamental frequency and thus is uncorrupted by higher harmonics. As a result, it is almost unaffected by the protophase-to-phase transformation [40], and it does not change (up to a constant phase shift) if we apply an invertible nonlinear transformation to the oscillatory component. For example, it will be the same for 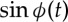 and 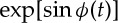, as well as for the respiration signals as simultaneously measured by belt and by thermistor, whereas the corresponding Hilbert phases will differ.

**Figure 2.**
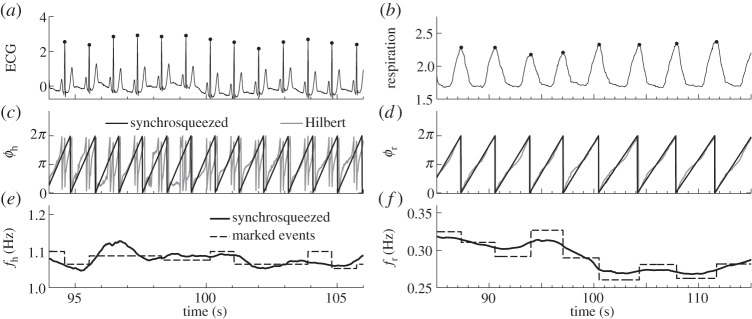
(*a*,*b*) The raw ECG and respiration signals with their peaks marked by dots. (*c*–*f*) The phases and frequencies of the cardiac (*ϕ*_h_,*f*_h_) and respiratory (*ϕ*_r_,*f*_r_) components, extracted from the signals by different methods. In (*c*,*d*), the phases extracted by the SWT are compared with those extracted by the Hilbert transform; the phases extracted by the ME method, apart from a constant phase shift in the ECG case, are almost indistinguishable from phases extracted by SWT within the resolution of the figure, and therefore are not shown. In (*e*,*f*), the frequencies extracted by SWT are compared with those found by the ME method; the frequency calculated from the Hilbert phase is usually inaccurate and seldom used. In both our cases, it deviates considerably from the SWT and ME frequencies.

Figure 2 shows typical phase and frequency results extracted by this method in comparison with the values extracted by other methods. The advantage over Hilbert phase extraction is clearly evident in figure 2*c*,*d*; in figure 2*c*, we illustrate the well-known fact that Hilbert phase extraction is inapplicable to the ECG, whose phase is usually obtained using the ME method. For clarity, the ME phase is not shown, but the difference is clearly visible in figure 2*e*,*f*, where the frequencies extracted by SWT and ME are compared. It should be noted, however, that one might apply the Hilbert transform to the signal after bandpass filtering in the region where the first harmonic resides, and also obtain a well-behaved phase; but the frequency calculated from it will not, in practice, be accurate. Moreover, when there are additional frequency components present, or where the main frequency varies by more than a factor of two (as is often the case for respiration), the frequency range of the first harmonic will inevitably contain additional components or harmonics. By contrast, SWT phase extraction uses a narrow but time-dependent adaptive frequency range, picking up only the main curve and thus avoiding the above problems.

**Figure 3.**
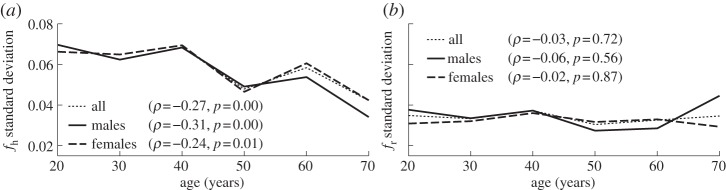
Age dependence of the standard deviations of (*a*) cardiac and (*b*) respiratory frequencies. Values of the correlation coefficient *ρ* and significance *p* (see appendix C) are indicated in each case.

Analysing the frequency extracted by SWT, one recovers the well-known result that heart rate becomes less variable with age [48–50]. Figure 3 shows how the standard deviation of the extracted frequency depends on age. Spearman's rank correlation coefficient *ρ* and its significance *p* (see appendix C) are also shown. From figure 3*a*, we see that the standard deviation of heart frequency has a negative correlation with age, i.e. the heart rate becomes less variable. There is no comparable effect in the case of respiration (figure 3*b*). The mean heart and respiratory frequencies do not change significantly with age (not shown), except for heart rate in males (*ρ*=−0.27, *p*=0.01).

## 3. Wavelet phase coherence

To investigate which oscillatory components are in common to both the respiration and heart activities, we calculated the WPC [27–31] between the IHF and the instantaneous respiration frequency (IRF), extracted from ECG and respiration SWTs, as well as between IHF and respiration. Note that the IHF is commonly, and somewhat confusingly, referred to in the literature [51] as HRV.

We calculated the WPC between the respiration signal and IHF, and between IRF and IHF. In doing so, we considered the frequency range 0.0095–2 Hz, which contains five physiologically relevant intervals [41]. To avoid bias owing to different lengths of physically relevant WT for different frequencies, we used the time interval of the WT corresponding to the lowest frequency (0.0095 Hz) for all scales. Note that, usually, one cannot properly consider the WT at frequencies greater than or approximately equal to 0.5 Hz for IHF (on account of Nyquist's theorem) because IHF extracted by the ME method has effective sampling frequency equal to the cardiac frequency (see above). In the present case, however, with SWT frequency extraction, we can go beyond this limit. Thus, in our case, the IRF and IHF have the physical sampling frequency of 50 Hz, so we can, in principle, go up to 25 Hz.

For oscillations with lower frequencies, there are fewer cycles within a given time interval. Their phase coherence is therefore expected to be larger, but without implying a higher level of interdependence. We therefore need to find an appropriate threshold (significance level) for each frequency, above which the phase coherence may be regarded as physically meaningful and indicates genuine interdependence.

To estimate significance levels, we use *intersubject surrogates*. Signals from different subjects must obviously be independent, while having similar characteristic properties. We therefore calculate surrogate values of, for example, the IHF–respiration phase coherence using respiration signal from one subject and the IHF from another. The significance level is then estimated as the 95th percentile of 189 surrogate values. Note that we use a significance level estimated by direct rank ordering (see [52, p. 352]) rather than based on the number of standard deviations (sigmas) above the mean of the surrogates, as is sometimes done. The latter criterion is not always appropriate because it implicitly assumes normally distributed surrogate values, which is untrue, in general, and for our case, in particular.

Use of other surrogate types, such as the widely used FT [52,53] or IAAFT [52,54] surrogates, is also possible. In our case, they may be used to test against the hypothesis that the apparent phase coherence obtained is entirely attributable to bias related to the frequency content and to the finite duration of the signal (and possibly to a distribution of values in the case of IAAFT). One might argue that our intersubject surrogates can sometimes have a lower significance level than (IAA)FT surrogates owing to the different breathing and cardiac frequencies for each subject. Although it appears not to be the case, it would not represent a problem even if true. We wish to test the presence/absence of dependence, rather than being interested in frequency bias in particular, so that the existence of exact matching of, for example, the mean frequency of respiration to the respiratory component in the IHF should not be neglected as is implicitly done by (IAA)FT. Thus, intersubject surrogates seem to represent the more natural choice for our case, as well as being convenient and computationally cheap. Nevertheless, given the high time-variability of cardiorespiratory signals, the WPC suffers bias only owing to the finite time length of the time series, so that (IAA)FT and intersubject surrogate tests become almost equivalent. We have found that they all give the same result in terms of significant WPC peaks.

**Figure 4.**
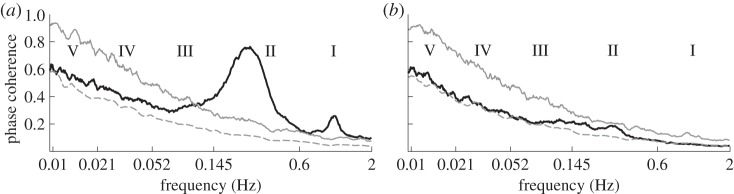
Wavelet phase coherence (WPC) as a function of frequency. The full black lines show the median (over all subjects) WPC between (*a*) respiration and instantaneous heart frequency (IHF) and (*b*) instantaneous respiration frequency (IRF) and IHF. The dashed grey lines indicate the mean of the surrogates, and the full grey lines show the surrogates' 95% significance level: the phase coherence is regarded as significant if it is higher than this. Vertical dotted lines indicate the boundaries of frequency regions corresponding to different physiological components [41, p. 74]: (I) cardiac, (II) respiratory, (III) myogenic, (IV) neurogenic, (V) NO-related endothelial.

Figure 4 shows the results for all subjects, but we have found that the same considerations apply to males and females separately. In the respiration–IHF phase coherence (figure 4*a*), there is a highly significant peak in the respiratory frequency range, corresponding to the well-known respiratory sinus arrhythmia (RSA). It was also observed previously in the cardiorespiratory coherence function, although the corresponding significance levels were not always estimated reliably (see [53] for discussion and references therein). We have observed a decrease with age in the respiratory peak WPC (not shown: *ρ*=−0.38 for males and *ρ*=−0.35 for females with *p*<0.001 for both). Coupled with the fact that the respiratory frequency and its standard deviation do not depend on age (figure 1*b*), this suggests that RSA becomes more unstable with age.

In the respiration–IHF WPC, there is also a significant peak at the cardiac frequency, which could not have been observed using the earlier ME- or IPFM-based methods of IHF extraction. It suggests that IHF and respiration share a cardiac component. Nevertheless, it is actually a measurement artefact, because the apparent cardiac component in respiration is attributable to the direct influence of heart beats on the respiratory measuring belt, which is not removed completely by the smoothing procedure (see appendix B). However, the presence of a cardiac component in the IHF is something new and interesting. It is clearly evident in the wavelet and SWTs of the IHF (not shown), but will not be discussed further here.

We find no significant coherence at all between the IHF and IRF (figure 4*b*). This implies that IHF and IRF do not, in general, share any frequency component, i.e. there is no common influence that measurably affects both the heart and respiration rhythms.

## 4. Model of cardiorespiratory interaction

We model the cardiorespiratory system as a pair of noisy coupled oscillators with phases *ϕ*_h,r_, where ‘h’ implies heart and ‘r’ implies respiration:
4.1
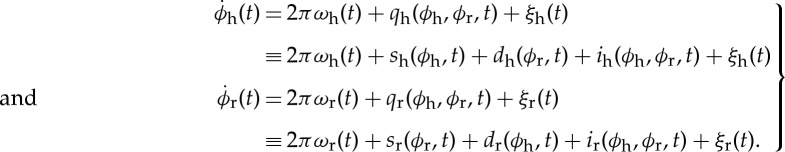
The time-derivatives of the phases 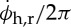 correspond to IHF and IRF, *ω*_h,r_ denote natural frequencies, and *ξ*_h,r_(*t*) is assumed to be white Gaussian noise: 〈*ξ*_*i*_(*t*)*ξ*_*j*_(*τ*)〉=*δ*(*t*−*τ*)*E*_*ij*_. The coupling functions *q*_h,r_ are separated into three parts: a self-interaction part *s*_h,r_ which accelerates/slows phase growth depending on the current phase of the oscillator considered; a direct interaction *d*_h,r_ which accelerates/slows phase growth depending on the current phase of the other oscillator; and an indirect part *i*_h,r_ which depends on both phases. Note that all terms are allowed to be time-dependent.

The self-interaction *s*_h,r_ reflects the effect of the oscillator on itself. In the case of using the protophase, for example, extracted by Hilbert transform, it could arise simply owing to nonlinearity, i.e. the contribution from the higher harmonics [40]. However, from the definition of phase, it follows that for the true phase the self-interaction can be only coupling-induced [40], being attributable to the interaction with other systems.

As implied by its name, the direct interaction *d*_h,r_ describes the direct influence, or driving of one oscillator by the other. We will refer to it as the ‘RSA-term’ because it is the only term that can be responsible for the stable respiratory modulation of IHF (and the corresponding phase coherence seen in figure 4*a*). This is because clear respiratory frequency modulation of 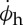 can be established only by the part dependent solely on *ϕ*_r_.

The last term, *i*_h,r_(*ϕ*_h_,*ϕ*_r_,*t*), describes a more complicated coupling, dependent on both phases. It is called indirect, because if the heart and respiratory activities both interact with some other system, for example, are each coupled to a third oscillator, this will be included in our model (4.1) as some complicated term(s) dependent on both heart and respiratory phases, and so be included within *i*_h,r_. Obviously, respiration and heart always interact through some other subsystems but, in the case of indirect coupling, we mean that the intermediate system is influenced by both respiratory and cardiac activity and does not merely transfer signals from one oscillator to the other. Nevertheless, the indirect interactions can also make a contribution to the direct and self-interaction parts. Such contributions are called coupling-induced and are mainly responsible for *s*_h,r_ in our case.

Let us illustrate the meanings of *s*_h,r_,*d*_h,r_,*i*_h,r_ by consideration of the mechanisms of cardiorespiratory interaction. It is well known that the respiratory centre modulates vagal motoneuron responsiveness (‘respiratory gating’) and thus heart rate [2,3]. Such ‘driving’ of 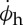 depends solely on the respiratory phase *ϕ*_r_, thus being completely included within the direct modulation term *d*_h_(*ϕ*_r_,*t*). Other influence on 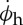 comes from the baroreflexes, which feel arterial pressure and induce heart rate changes in response to its variations [55]. Thus, heart, arterial pressure and respiration interact in a closed loop [56,57]. Because arterial pressure is modulated by both heart and respiration [58], depending on both *ϕ*_h,r_, this mechanism will be included mainly within the indirect term *i*_h_(*ϕ*_h_,*ϕ*_r_,*t*). As already mentioned, it can contribute to *s*_h,r_,*d*_h,r_ as well.

Our model (4.1) includes explicitly only the phases of the cardiac and respiratory activities. In general, IHF and IRF are modulated by other processes as well, for example, modulation of IHF at low frequencies around 0.1 Hz [5,35,48–50,56]. Nonetheless, such additional mechanisms are effectively accounted for by allowing the terms in (4.1) to be time-dependent. At the same time, consideration of other signals such as arterial pressure can provide additional insights into the underlying dynamics. Thus, in [57], the authors studied in addition arterial pressure, making it possible to separate the influence of respiration on IHF into the effect owing to ‘respiratory gating’ and that owing to baroreflex coupling with arterial pressure fluctuations (modulated in part by respiration). Here, however, we are mainly interested in the age dependence of the coupling parameters and not so much in revealing the physiological origins of the interactions.

## 5. Extraction of nonlinear interactions using Bayesian inference

The phase dynamics of cardiorespiratory interactions can be analysed by application of a technique based on Bayesian inference [59–62]. To extract the parameters of interaction (*ω*_h,r_,*q*_h,r_), we apply the Bayesian method extended to incorporate time variability [37,38] to the phases extracted from the SWT. Thus, we model the right-hand sides of (4.1) as a sum of chosen basis functions multiplied by some coefficients 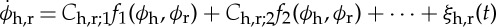, and find most probable values of these coefficients *C*_h,r;*i*_ in each time window.

Because all functions of phase, such as *q*_h,r_(*ϕ*_h_,*ϕ*_r_,*t*), should be 2*π*-periodic over each phase, the most appropriate basis functions are Fourier series. We used 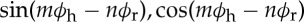 with *n*=−2, −1, 0, 1, 2 and *m*=−2, −1, 0, 1, 2, i.e. Fourier series up to second order. In addition, we used synchronization terms with *n*:*m*=3:1, 4:1, 5:1, 6:1, 7:1, 7:2, 9:2, 10:3, corresponding to the common cardiorespiratory synchronization ratios [63,64]. Altogether, for each of 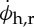, we used 41 basis functions. We used non-overlapping windows of time length 50 s, chosen to incorporate at least 10 respiratory cycles and thus provide enough information for accurate inference. We perform inference within each window, so all parameters are determined on this time scale (e.g. the synchronization duration is ‘quantized’ in units of 50 s). The propagation constant [38] was chosen by trial-and-error basis to be *p*_w_=20. Its significance relates to the propagation of information, i.e. the extent to which inference in the current time window takes account of the result obtained in the previous one. For completely independent inference in each window, 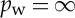, whereas *p*_w_=0 means full propagation. The choice of *p*_w_ does not significantly influence the results.

With the chosen basis functions, the self-interaction (*s*_h,r_) and direct interaction (*d*_h,r_) parts of the coupling function are modelled using 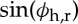, 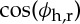, 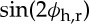, 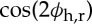 (*ϕ*_h_ for *s*_h_,*d*_r_ and *ϕ*_r_ for *s*_r_, *d*_h_), whereas all other basis functions except unity (for *ω*_h,r_) are included within the indirect interaction *i*_h,r_. Thus, the model for *s*_r_ and *d*_h_ can be represented in the form
5.1
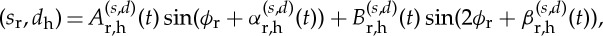
and similarly (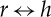) for *s*_h_,*d*_r_. From this form, it is clear that only the direct and self-interaction terms can be responsible for possible cardiac and respiratory components and their harmonics in the IHF and IRF; all other terms depend on both phases and thus generally cannot have purely respiratory or cardiac frequency. It should be noted that incorporation of higher terms, such as Fourier series up to fourth order, does not lead to results qualitatively different from those presented below.

### (a) Synchronization

Having determined for some window the most probable coefficients *C*_h,r;*i*_ multiplying the basis functions, we can find out whether there is *n*:*m* synchronization between *ϕ*_h_ and *ϕ*_r_ within that window. To do so, we first fix *ψ*_*n*,*m*_≡*ϕ*_h_/*n*−*ϕ*_r_/*m*, and for each value integrate (4.1) with inferred right-hand sides, so that *ϕ*_h_/*n*+*ϕ*_r_/*m* go from 0 to 2*π*. Let us denote the value of *ϕ*_h_/*n*−*ϕ*_r_/*m* at the end of this procedure as *M*(*ψ*_*n*,*m*_), because it depends on the initial choice of *ψ*_*n*,*m*_. Then, most probably there is synchronization if there exists a *ψ*_*n*,*m*_ such that *M*(*ψ*_*n*,*m*_)=*ψ*_*n*,*m*_ and, for synchronization to be stable, |d*M*(*ψ*_*n*,*m*_)/d*ψ*_*n*,*m*_|<1. If there is such a root, then we consider there to be synchronization throughout the duration of the selected window, and otherwise no synchronization within that window.

A major advantage of the Bayesian approach to the investigation of synchronization is that it is more noise robust [38] than methods based on the synchrogram [64] or on different synchronization indices [65–67]. This is because through the inference of noise parameters, one can explore the uncorrupted underlying dynamics. A detailed comparison of approaches is a separate topic, but we comment that we obtained qualitatively the same results by the application of the phase coherence method [65].

**Figure 5.**
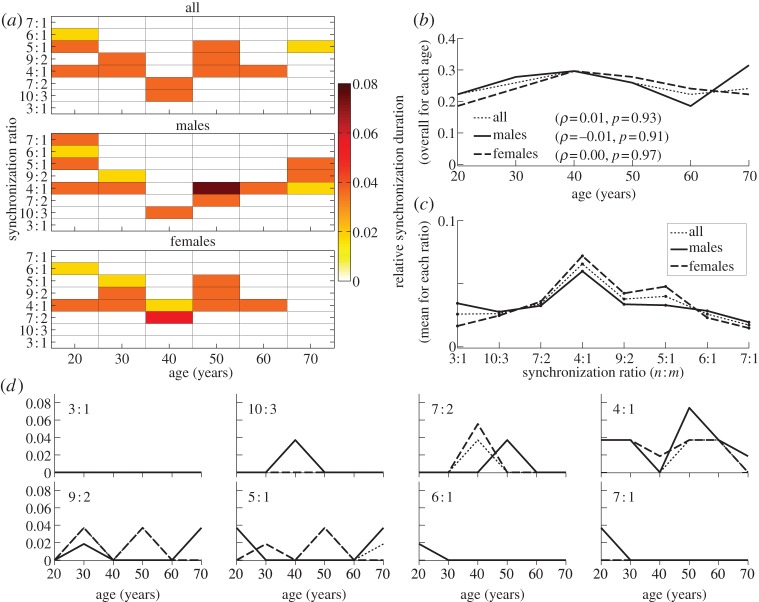
Age dependence of the relative duration of cardiorespiratory synchronization, i.e. the proportion of the measurement time within which there was synchronization at particular ratios: (*a*) summary of results for each *n*:*m* synchronization ratio, age and gender; (*b*) overall synchronization duration, i.e. the sum of durations for each ratio; (*c*) average synchronization duration at each ratio, taken over all males, all females and the merged group; note that it is the mean, unlike the median in all other panels: the corresponding median is zero for all ratios except 4:1; (*d*) the same as (*a*), but for each synchronization ratio separately; *ρ* and *p* are not shown, but there are no significant monotonic correlations for any ratio. (Online version in colour.)

Figure 5*a* summarizes all the information about synchronization obtained in this way, in the form of the relative synchronization duration, i.e. the proportion of the time during which the signals were synchronized at a particular *n*:*m* ratio. Note that, because synchronization is quantized in 50 s intervals, the results do not take into account possible shorter episodes of synchronization. Therefore, in general, the relative synchronization duration will actually be higher. Second, the calculated overall synchronization duration relates only to the particular synchronization ratios considered, taking no account of, for example, 9:1 synchronization. As can be seen, however, even the 6:1 and 7:1 ratios are quite rare, whereas the most prevalent ratio is usually 4:1 (figure 5*c*).

No monotonic age dependence of the overall degree of synchronization has been found (figure 5*b*), and nor has it for synchronization at any particular ratio (not shown). Nevertheless, it seems that certain synchronization ratios may be age- and gender-dependent, for example, 10:3 synchronization arises mainly in males aged around 40 (figure 5*a*,*d*). However, a more detailed investigation, involving larger numbers of subjects of given age and gender, would be needed to draw strong conclusions about particular age/gender groups (see appendix C).

### (b) Directionality

We calculate the directionality of influence as 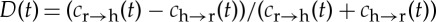, where 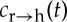 is calculated as the norm of the coefficients (
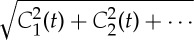
) of the basis functions that depend on *ϕ*_r_ (i.e. correspond to *d*_h_ and *i*_h_) and represents the influence of respiration on the heart; correspondingly, 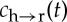 represents the influence of the heart on respiration.

**Figure 6.**
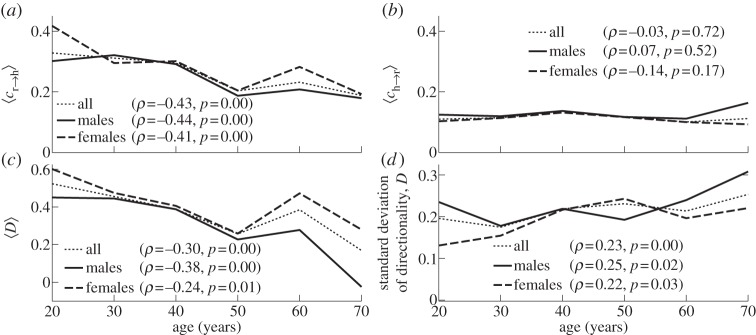
Evolution with age in the strength and direction of cardiorespiratory influence. (*a*) Influence of respiration on the heart, 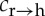. (*b*) Influence of the heart on respiration, 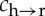. (*c*) The net directionality of coupling, *D*, from respiration to heart. (*d*) Standard deviation of the directionality of coupling. In (*a*–*c*), the time-averages are shown.

Figure 6 shows the dependence on age of the time-averaged 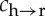, 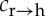 and the resultant coupling directionality *D* together with its standard deviation. It is clear from figure 6*a* that the influence of respiration on the heart has significant negative correlation with age, whereas the influence of the heart on respiration is not age-dependent as shown in figure 6*b*. As a result, the time-average of coupling directionality significantly decreases with age (figure 6*c*). At the same time, its standard deviation increases with age (figure 6*d*), implying that the cardiorespiratory interaction also becomes less stable.

To ascertain whether the decrease of influence of respiration on heart (and thus directionality) reflects only a decrease of RSA, or whether it implies something more, we now consider the relative contributions made by different terms in the directionality. Let us denote by 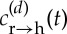 the norm of the coefficients multiplying the basis functions in *d*_h_, i.e. representing the RSA. Similarly, we define 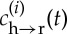 as the norm of the coefficients in *i*_h_, representing the contribution from the indirect interactions. We do the same for *d*_r_,*i*_r_.

**Figure 7.**
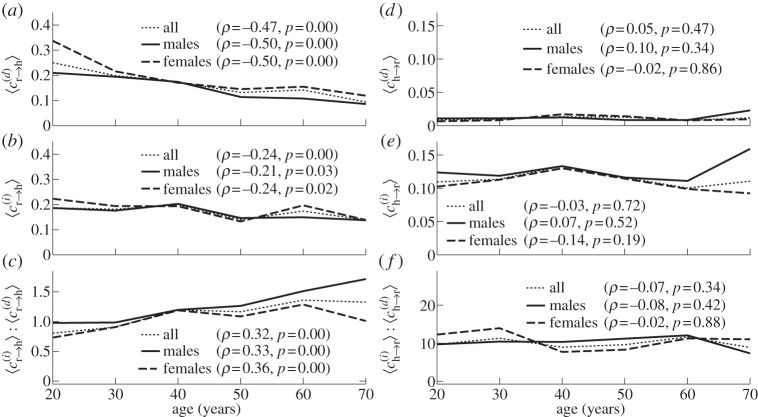
(*a*–*f*) Dependence on age of the different contributions to coupling directionality (see text).

Figure 7 shows time-averages of the different contributions to the cardiorespiratory interaction, as well as their mutual relationships. It is evident that both the direct and indirect influences of respiration on the heart are negatively correlated with age (figure 7*a*,*b*), with the direct contribution decreasing faster than the indirect one (figure 7*c*). At the same time, contributions to the influence of the heart on respiration and the corresponding indirect/direct ratio exhibit no significant age dependence (figure 7*d*–*f*). Thus, the decreases with age of 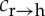 and coupling directionality are completely attributable to the combined effects of a decrease in RSA and the indirect effect of respiration on the heart.

We also analysed in detail the direct and self-interaction terms (5.1), characterizing their shape variations by the amplitude ratio *B*(*t*)/*A*(*t*) between two harmonics in (5.1). We found a significant increase with age in the time-averaged 〈*B*/*A*〉 ratio (*ρ*=0.38,*p*=0.00 for males and *ρ*=0.29,*p*=0.01 for females), implying that the form of RSA becomes less harmonic with age. We also found a significant increase in the standard deviation of this ratio (males: *ρ*=0.34,*p*=0.00; females: *ρ*=0.28,*p*=0.01) and a decrease of the phase coherence *γ*=|〈e^i(2*α*(*t*)−*β*(*t*))^〉| between harmonics in (5.1) (males: *ρ*=−0.29,*p*=0.00; females: *ρ*=−0.33,*p*=0.01). This indicates an increase in RSA time-variability with age, consistent with the result of figure 6*d*, and a decrease in respiratory WPC with age (see §3). No significant correlations with age were observed in the cases of *s*_h_,*s*_r_,*d*_r_.

### (c) Coupling function

**Figure 8.**
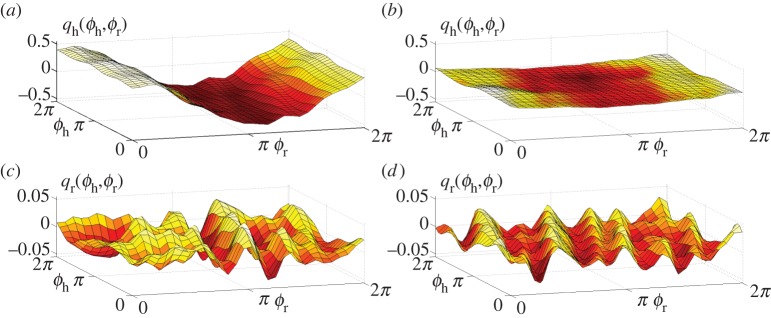
Typical time-averaged coupling functions in (4.1) for (*a*,*c*) a young and (*b*,*d*) an old male subject, aged 21 and 71 years, respectively. Their time evolutions can be viewed as videos (see footnote 1). (Online version in colour.)

To gain further insights into the nature of the cardiorespiratory interaction, we now analyse the overall form of the reconstructed coupling functions *q*_h,r_(*ϕ*_h_,*ϕ*_r_,*t*) (4.1). Figure 8 shows the time-averaged versions of the coupling functions *q*_h,r_ typical of a younger and an older subject. Decrease of the RSA amplitude with age is clearly seen in figure 8*a*,*b*. It can also be concluded that the main stable contribution to *q*_h_, surviving after time-averaging, remains the RSA irrespective of age. The respiratory coupling *q*_*r*_ shown in figure 8*c*,*d* seems to be quite irregular and not age-dependent.

However, the time-averaged coupling functions provide only limited information. The full time evolution of the same coupling functions are illustrated through videos.^1^ From them, one can see that, in older people, the heart coupling function becomes also less stable in time, dominated by the highly time-variable indirect contributions. At the same time, the dynamics of *q*_*r*_ does not seem to change with age, being irregular and unstable.

## 6. Summary and conclusions

Our investigations have yielded results in agreement with those reported earlier [41,48–50], while also revealing many new features that invite a reconsideration of some of the phenomena observed previously. In particular are the following.

— *Frequencies*. Applying the SWT [39] to measured time series, we have obtained the physically relevant instantaneous frequencies of the heart (IHF, commonly referred to as HRV in the literature) and respiration (IRF) with a sampling frequency of 50 Hz, as well as their corresponding phases. In agreement with earlier works [41,48–50], we observed a decrease of IHF variability with age (figure 3*a*), but no significant correlation with age in IRF variability (figure 3*b*) or mean respiratory frequency (not shown).
— *Coherence*. Computing the WPC (§3) and estimating its significance threshold by the use of intersubject surrogates, we found statistically significant peaks at both the respiratory and heart frequencies in the respiration–IHF WPC (figure 4*a*). The former is attributable to RSA and is in agreement with earlier findings [53], whereas the latter represents a measurement artefact (see appendix B). There is also a decrease with age in the respiration–IHF phase coherence at respiratory frequencies, indicating that RSA becomes less stable. There is no significance in the IRF–IHF WPC (figure 4*b*), indicating that the heart and respiratory rates are modulated mainly by separate mechanisms.
— *Bayesian inference*. We model the cardiorespiratory system as a pair of noisy coupled oscillators with explicitly time-dependent coupling parameters. Heart and respiratory coupling functions were divided into three parts, representing: coupling-induced self-interaction; direct driving by the other system; and indirect interactions. The direct influence of respiration on the heart was attributed to ‘respiratory gating’ [3], i.e. RSA, whereas the indirect influence was related to baroreflex coupling [55] with possible contributions from other mechanisms. By reconstructing the model equations and fitting them directly to the measured time series [38,59–62], we have obtained the forms and time evolutions of the underlying heart and respiratory coupling functions (figure 8, see also footnote 1), confirming and extending the preliminary results of [38]. They were then used to analyse synchronization, directionality of coupling and the contributions of different mechanisms to the cardiorespiratory interaction.
— *Synchronization*. There is no significant correlation of the overall synchronization duration with age (figure 5*b*), consistent with recent findings [64] based on the synchrogram method. The most common cardiorespiratory synchronization ratio was found to be 4:1 (figure 5*c*,*d*). It seems also that certain synchronization ratios may be characteristic of particular ages and genders (figure 5*a*,*d*).
— *Coupling directionality*. The overall influence of respiration on the heart decreases with age (figure 6*a*), whereas influence in the opposite direction stays constant (figure 6*b*), leading to a net decrease of coupling directionality with age (figure 6*c*). Directionality also becomes more time-variable with age (figure 6*d*), corresponding to a progressive impairment of the underlying mechanisms.
— *Cardiorespiratory interactions*. Consistent with earlier findings [41,48–50] the RSA amplitude, i.e. the strength of the direct respiratory modulation of heart rate, decreases with age (figure 7*a*), as well as changing form and becoming more time-variable (see last paragraph of §5*b*). Because the breathing rate does not change with age, these effects cannot be attributable to the influence of the breathing frequency on RSA [68,69]. The indirect modulation of the heart by respiration also decreases with age (figure 7*b*), though not so rapidly as RSA (figure 7*c*). This effect may correspond to a decrease in baroreflex sensitivity with age [70,71]. The strengths of the direct and indirect influences of heart on respiration (figure 7*d*,*e*) do not change with age.
— *Coupling functions*. Underlying all these separate effects, the heart coupling function changes markedly with age, both in its average form (figure 8*a*,*b*) and in its time-variability (see footnote 1), whereas the respiratory coupling function seems to be irregular and unaffected by age (figure 8*c*,*d*, and see footnote 1).

## Figures and Tables

**Figure 9.**
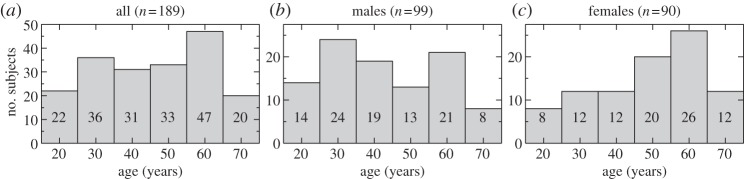
Age distribution of the subjects, shown as an overall histogram (*a*) and then divided by gender (*b*,*c*).

## Footnotes

1 Videos of time evolution of cardiorespiratory coupling functions for the same subjects as used in figure 8 can be seen at http://www.physics.lancs.ac.uk/research/nbmphysics/diats/crt.

## Appendix A. Subjects and measurements

The subjects included 102 males (43.1±16.3 years, range 16–77 years) and 95 females (48.8±16.2 years, range 18–90 years) with no prior history of cardiovascular disease. For each subject, a conventional three-lead ECG signal was measured with electrodes on both shoulders and on the lowest left rib, and an elastic belt with an attached Biopac TSD201 respiratory effort transducer (Biopac Systems Inc., CA, USA) was positioned around the chest to record a respiration signal. The signals were recorded continuously and simultaneously for 30 min with subjects lying relaxed and supine in a quiet environment at normal room temperature. For the first 113 recordings [41], initial signal amplification was performed using a specially designed signal conditioning unit (Cardiosignals, Jožef Stefan Institute, Ljubljana, Slovenia), and the signals were digitized at 400 Hz by use of a 16-bit A/D converter. The subsequent 84 recordings [32] were amplified with an updated signal conditioning unit (Cardio&Brain Signals, Jožef Stefan Institute) and digitized at 1200 Hz using a 24-bit A/D converter.

Subjects gave their written consent, and the study was approved by the UK National Research Ethics Service. All measured subjects had systolic blood pressures less than 150 mm Hg and diastolic blood pressures less than 90 mm Hg, body mass indices less than 30, and skin temperatures more than 28.5^°^C during the recording.

## Appendix B. Filtering and pre-processing

After signals with significant movement artefacts or frequent ectopic beats (more than 50 during the 30 min recording) had been discarded, we were left with 189 sets of data (99 from males, 90 from females). The ECG and respiration signals from each subject were pre-processed as follows. The first and last 1 per cent of each signal were discarded to eliminate the starting and ending transients present in some of them. Next, those signals sampled at 1200 Hz were resampled to our standard 400 Hz. All signals were then detrended using a 200 s window. The belt used to measure respiration ‘feels’ the heart beats directly, and consequently introduces a spurious heart component into the respiration signal. This effect can be clearly observed during a breath hold. To partly eliminate it, the respiratory signals were smoothed by use of a 1 s moving average. All signals were then resampled to 50 Hz, matched in length, and de-meaned. These procedures were applied carefully, in such a way as to preserve the exact time relationship between each pair of signals.

## Appendix C. Statistical methods

To quantify the significance of the observed monotonic age dependence, we have used Spearman's rank correlation coefficient *ρ* (see [41], §C.2.2), and (two-tailed) statistical significance was assumed for values of *p*<0.05. The correlation coefficient for all males, all females and the merged group, as well as its significance, is presented in each figure rounded to two decimal places. For example, ‘*p*=0.00’ implies *p*<0.005.

Because the Spearman's correlation coefficient only takes account of monotonic dependence, the subjects' datasets were divided into age bins, and medians over each bin are shown in the figures in order to better eliminate possibly nonlinear age dependence. The age bins are centred on 20, 30, 40, 50, 60 and 70 years and, except for the last group, are each of 10 years duration (e.g. the 50-year group includes subjects aged 46–55). The final bin includes all subjects older than 65 (up to 90). Age distributions for each gender are presented in figure 9. Note, however, that comparisons between age bins of particular gender may have not enough subjects for good statistics, given the complexity of the cardiorespiratory system and large individual influences. Therefore, our main conclusions are all based on the Spearman's correlation and its significance, which take into account all 99 male and/or 90 female subjects, whereas the information over age bins is provided mainly as a guide to the eye.
